# Association between Selected Polymorphisms rs12086634, rs846910, rs4844880, rs3753519 of 11β-Hydroxysteroid Dehydrogenase Type 1 (*HSD11B1*) and the Presence of Insulin Resistance in the Polish Population of People Living in Upper Silesia

**DOI:** 10.3390/ijerph181910168

**Published:** 2021-09-27

**Authors:** Nikola Szweda-Gandor, Mirosław Śnit, Władysław Grzeszczak

**Affiliations:** Department of Internal Medicine, Diabetology and Nephrology, Faculty of Medical Sciences in Zabrze, Medical University of Silesia, 40-155 Katowice, Poland; nszweda@sum.edu.pl (N.S.-G.); msnit@sum.edu.pl (M.Ś.)

**Keywords:** selected polymorphism, *HSD11B1*, insulin resistance

## Abstract

Background: Many factors influence the development of insulin resistance, among other genetic factors. Cortisol is one of the factors that has a significant impact on the development of insulin resistance. The proteins that have a substantial effect on blood cortisol levels include 11β-hydroxysteroid dehydrogenase type 1. *HSD11B1* is a microsomal enzyme that catalyzes the conversion of the stress hormone cortisol to the inactive metabolite cortisone. Gene encoding *HSD11B1* is located on 1q32.2. This study was designed to assess the association between four polymorphic sides in *HSD11B1* (rs12086634, rs846910, rs4844880, rs3753519) between subjects with and without insulin resistance in the Polish population of people living in Upper Silesia. Methods: The study included a total of 507 consecutive patients, 374 (73.77%) with and 133 (26.23%) without insulin resistance. Results: The results show that there were no statistically significant differences in the distribution of genotypes and alleles of the examined polymorphisms of the 11β-hydroxysteroid dehydrogenase type 1 gene between subjects with and without insulin resistance (determined using the HOMA-IR, insulin resistance index) and that rs846910 and rs1208663 polymorphisms of the 11β-hydroxysteroid dehydrogenase type 1 gene in the examined subjects have a significant effect on the magnitude of the HOMA-IR insulin resistance index. Conclusions: The study results suggested that genetic variation of rs846910 and rs1208663 polymorphism of the *HSD11B1* gene is related to the susceptibility to insulin resistance. Our results provide a basis to begin basic research on the role of the *HSD11B1* gene in the pathogenesis of insulin resistance.

## 1. Introduction

Insulin resistance is a condition of decreased tissue sensitivity to insulin. Insulin resistance may be primary (due to inheritance of a genetic mutation) or secondary [1,2,3,4]. Increased insulin resistance increases indirectly the incidence of myocardial infarction, stroke, peripheral arteriosclerosis, and increased blood pressure [5,6,7]. The most common conditions in which insulin resistance is found are type 2 diabetes, primary hypertension, and renal failure. Insulin resistance is a major component of the metabolic syndrome [8,9,10,11,12]. So far, the mechanism of development of insulin resistance has not been clearly established [13,14]. Many factors influence the development of insulin resistance, including genetic factors [12,15,16]. Cortisol is one of the factors that has a significant impact on the development of insulin resistance [17]. Too much cortisol can lead to central obesity, and a particular variation in this gene has been associated with obesity and insulin resistance [15,18]. Among the proteins that have a significant effect on blood cortisol levels, there is 11β-hydroxysteroid dehydrogenase type 1 [19]. *HSD11B1* is a microsomal enzyme that catalyzes the conversion of the stress hormone cortisol to the inactive metabolite cortisone [20]. In addition, the encoded protein can catalyze the reverse reaction, the conversion of cortisone to cortisol [21]. Gene encoding *HSD11B1* is located on 1q32.2, has seven exons and around nine thousand base pairs [22]. In our paper we analyzed four polymorphic sides in this gene (rs12086634, rs846910, rs4844880, rs3753519).

## 2. Aim

The aim of this study is to find answers to the following questions:

1. Are there any significant differences in the distribution of genotypes and alleles of selected polymorphisms of the 11β-hydroxysteroid dehydrogenase gene (rs12086634, rs846910, rs4844880, rs3753519) between subjects with and without insulin resistance?
2. Are there any significant differences in the distribution of genotypes and alleles of selected polymorphisms of the 11β-hydroxysteroid dehydrogenase gene (rs12086634, rs846910, rs4844880, rs3753519) between male and female subjects with and without insulin resistance?
3. Is there any association between the studied polymorphisms of the 11β-hydroxysteroid dehydrogenase (rs12086634, rs846910, rs4844880, rs3753519) and the HOMA-IR coefficient?

## 3. Patients and Methods 

### 3.1. Patients

The study included a total of 507 consecutive patients who randomly attended the general outpatient clinic of the private health care institution “Gmin-Med” in Dobieszowice (Poland). The group consisted of 223 men (44%) and 284 (56%) women, the presence of insulin resistance was detected in 374 (73.77%) of the study samples (study group), while 133 (26.23%) of the patients did not have insulin resistance (control group).

Participation in the study was offered to every adult patient who came to the outpatient clinic “Gmin-Med”. During the appointment, the patients who agreed to participate in the medical experiment were asked to answer the research questionnaire. The data provided were verified based on the available medical records. After obtaining written informed consent to participate in the clinical trial, venous blood was taken at the local laboratory, belonging to the “Gmin-Med”—venous blood was collected for biochemical, hormonal, and genetic studies.

Basic information about the subjects is shown in Table 1.

There was a statistically significant difference in HOMA-R values between subjects (women and men) with and without insulin resistance (*p* < 0.001).

### 3.2. Ethical Approval

The study protocol was approved by the Bioethics Committee of the Medical University of Silesia (Resolution 20/2010). The research was conducted in line with the Declaration of Helsinki. All participants provided written informed consent to take part in the study.

### 3.3. Methods

Approximately 20 mL of venous blood was collected from each subject in the morning on fasting. The test material was obtained using a Sarstedt S-Monovette closed aspiration-vacuum blood collection system. Two blood samples of 4.9 mL each were collected into tube-syringes with EDTA as anticoagulant and frozen at −20 °C, and this material was later used for genetic studies. After centrifugation, the serum from one tube was used for biochemical tests performed at the local laboratory, and the serum from the other tube was frozen at −20 °C. 

Advanced biochemical, hormonal and genetic tests were performed in the laboratory belonging to the Department of Internal Medicine, Diabetology and Nephrology, Medical University of Silesia. Insulin and blood glucose levels of the patients were determined. Insulin concentration was determined by Insulin DRG ELISA. Glucose concentration was determined using Epol 20 Bio spectrophotometer. Measurements of insulin and glucose concentrations were used to assess insulin resistance.

The HOMA-IR insulin resistance index was used in the study. Insulin resistance was diagnosed based on the results of the HOMA-IR test. The HOMA-IR is calculated using the following formula: fasting blood insulin level [mU/mL] × fasting blood glucose level [mmol/L] /22.5. We diagnosed insulin resistance when the HOMA-IR was > 2.0. 

In the 11β-hydroxysteroid dehydrogenase type 1 gene (*HSD11B1*), we examined the distribution of the following polymorphisms: *HSD11B1* rs12086634, *HSD11B1* rs846910, *HSD11B1* rs4844880, *HSD11B1* rs3753519. Genotyping of polymorphisms was performed with fluorescently labeled probes using the TaqMan Pre-designed SNP Genotyping Assay single nucleotide polymorphism (SNP) kit (Applied Biosystems, Waltham, MA, USA). Polymerase chain reaction (PCR) and allele identification were performed on a 7300 Real Time PCR System (Applied Biosystems).

### 3.4. Statistical Analysis

To identify significant differences between groups, the t-test was used for variables with normal distribution and the Mann–Whitney test was used for variables without normal distribution. The genotype distribution was compared between groups using the χ^2^ test with Yates correction. A *p*-value of 0.05 or less was considered significant. Statistical analysis was performed using Microsoft Excel 2013 (Microsoft Corporation, Redmond, WA, USA) and Statistica 13.1 (StatSoft Inc., Tulsa, OK, USA). 

## 4. Results

Table 2 shows the results of the distribution of genotypes tested rs12086634, rs846910, rs4844880, rs3753519 of the 11β-hydroxysteroid dehydrogenase type 1 (*HSD11B1*) gene in the entire study group of subjects, in women with and without insulin resistance, and in men with and without insulin resistance.

We did not find any statistically significant differences in the distribution of rs12086634, rs846910, rs4844880, rs3753519 genotypes of the *HSD11B1* gene in subjects with and without insulin resistance (all groups with/all groups without, all women/all men, women with/women without and men with/men without).

Table 3 shows the results of the distribution of alleles tested rs12086634, rs846910, rs4844880, rs3753519 of the 11β-hydroxysteroid dehydrogenase type 1 (*HSD11B1*) gene in the entire study group of subjects, in women with and without insulin resistance, and in men with and without insulin resistance. 

We did not find any statistically significant differences of alleles distribution tested rs12086634, rs846910, rs4844880, rs3753519 of *HSD11B1* gene subjects with and without insulin resistance (all groups with/all groups without, all women/all men, women with/women without and men with/men without) despite significant differences between men with and without insulin resistance in rs1208663 (*p* = 0.03488).

Table 4 shows the mean HOMA-IR values for the different polymorphisms of the type 1 *HSD11B1* gene in the entire study group of subjects, in women with and without insulin resistance, and in men with and without insulin resistance.

The data presented in Table 4 show that the value of the HOMA-IR coefficient was significantly higher in subjects with GG genotype rs846910 compared to AG and AA rs846910 genotypes and it can be observed in all the studied groups except two groups—women and men without insulin resistance. Similarly, the value of the HOMA-IR coefficient was significantly higher in the subjects with GG rs1208663 genotype in comparison to AG and AA rs1208663 genotypes in all the groups except for subjects with insulin resistance. The HOMA-IR value was only higher in subjects with TT rs3753519 and rs4844880 genotype relatively to CC and CT rs3753519 genotypes and AA and AT rs4844880 genotypes.

## 5. Discussion

In recent years, there has been a significant increase in the number of individuals diagnosed with insulin resistance. Insulin resistance is accompanied by a significant ongoing increase in the risk of metabolic disorders, including carbohydrate abnormalities [19]. Insulin resistance alone can lead to an increased risk of type 2 diabetes, cardiovascular disease, inflammation, polycystic ovarian syndrome, hepatic steatosis, and other disorders even in individuals without obesity.

Both genetic and environmental factors play an important role in the pathogenesis of insulin resistance development [19]. All known factors affecting insulin sensitivity—including obesity and other environmental factors such as poor diet, physical inactivity, alcohol, and stress—explain less than one-third of the variation in insulin sensitivity in the population. It is possible that genetic factors may play an important role in insulin resistance.

Therefore, in our study we attempted to assess the significance of four selected polymorphisms of the 11β-hydroxysteroid dehydrogenase type 1 gene (rs12086634, rs846910, rs4844880, rs3753519) in the pathogenesis of insulin resistance development/occurrence.

In order to find an answer to the questions posed in the aim of this study, we performed more than 2000 determinations of the four specific polymorphisms of the 11β-hydroxysteroid dehydrogenase type 1 gene in a group of 507 patients, consecutively attending an outpatient clinic in Dobieszowice, Upper Silesia (Poland). We determined polymorphisms rs12086634, rs846910, rs3753519, rs4844880 c in blood samples selected from the subjects. This gene is located on chromosome 1 (the largest human chromosome), in introns (rs3753519 at position: 209702170, rs4844880 at position: 209697571, rs12086634 at position: 209,706,914 and rs846910 in item: 209701909).

Our results showed that there was no statistically significant difference in the distribution of rs12086634, rs846910, rs4844880, rs3753519 genotypes of the *HSD11B1* gene in subjects with and without insulin resistance. In contrast, we showed a statistically significant difference in the distribution of rs 1,208,663 alleles between men with and without insulin resistance.

Other authors have also studied the association between the presence of metabolic syndrome or type 2 diabetes and polymorphisms of the *HSD11B1* gene, especially the rs846910 polymorphism. We would like to emphasize again that in the pathogenesis of metabolic syndrome or type 2 diabetes, the presence of insulin resistance is important. The authors Devang et al. [2] showed that the presence of the G allele rs846910 of the *HSD11B1* gene is accompanied by a lower risk of developing diabetes and metabolic syndrome. Other authors studying this issue showed that the presence of the G rs846910 allele of the *HSD11B1* gene is accompanied by a higher risk of developing diabetes and metabolic syndrome.

The other polymorphisms of the *HSD11B1* gene that we studied (rs12086634, rs4844880, rs3753519) have not yet been the subject of other detailed studies. Therefore, we cannot refer to other research results.

In our study, we showed that the value of the HOMA-IR coefficient was significantly higher in subjects with GG genotype rs846910 compared to subjects with AG and AA genotypes rs846910 and this was in all the studied groups except two groups, women and men without insulin resistance. Similarly, the HOMA-IR value was significantly higher in subjects with GG rs1208663 genotype compared to AG and AA rs1208663 genotypes in all study groups except in subjects with insulin resistance. The HOMA-IR value was higher only in subjects with TT rs3753519 and rs4844880 genotypes compared to CC and CT rs3753519 and AA and AT rs4844880 genotypes (Table 4).

As reported in the literature, genetic variants in the *HSD11B1* gene are promising contributors to type 2 diabetes (T2D) and metabolic syndrome (MetS) [23,24,25]. *HSD11B1* rs846910 affects transcription by acting as a heat shock transcription factor binding site [23]. Interesting observations have been made in different populations regarding the association of HSD11B1 gene variants with T2D, [23] and MetS [24,25].

Thus, Devang et al. [2] in an Indian population evaluated, among others, the association of the rs846910 polymorphism of the *HSD11B1* gene with type 2 diabetes and metabolic syndrome. In an association analysis, they showed that HSD11B1 rs846910 A contributes to an increased risk of developing T2D (OR = 1.62; 95% CI 1.02–2.57, *p* = 0.03). It is only appropriate to mention at this point that insulin resistance plays a key role in the pathogenesis of T2D development.

The authors Gasparin et al. [26] studied the association of the presence of the G allele rs846910 of the *HSD11B1* gene with overweight and obesity in children and adolescents. The authors showed that the G allele (rs846910) of the *HSD11B1* gene was significantly statistically associated with being overweight (*p* = 0.039). In conclusion, the authors suggest that the G (rs846910) allele of the HSD11B1 gene may be a risk factor for being overweight. It should not be forgotten at this point that insulin resistance is important in the pathogenesis of obesity development.

On the other hand, authors Quteineh et al. [5] studied the relationship between seven polymorphisms of the *HSD11B1* gene, and BMI and MetS components in a group of psychiatric patients treated with psychotropic drugs. These drugs potentially cause weight gain (n = 478). The presence of the *HSD11B1* rs846910-A allele was associated with lower BMI, lower waist circumference, and lower diastolic blood pressure compared to subjects with the rs846910-G genotype present. In conclusion, the authors write that certain *HSD11B1* polymorphisms, including rs846910, may contribute to the development of MetS in psychiatric patients receiving psychotropic treatment.

The aim of the study conducted by the authors Farag et al. [27] was to determine the effect of the rs846910 polymorphism of the *HSD11B1* gene in patients with metabolic syndrome. The authors showed that subjects with the rs846910 GG genotype were significantly associated with increased body mass index (BMI), increased cholesterol, increased low-density lipoprotein (LDL), increased cortisol, and decreased high-density lipoprotein. As before, we would like to emphasize that subjects with metabolic syndrome most often have insulin resistance.

In our study we also showed an association between the presence of rs846910 polymorphism of the *HSD11B1* gene and the risk of insulin resistance. Other authors have also focused their attention on conducting studies in this direction.

Devang et al. [2], among others, evaluated the association of rs1208663 polymorphism of the *HSD11B1* gene with type 2 diabetes and metabolic syndrome. In an association analysis, they showed that *HSD11B1* rs12086634 TG contributes to an increased risk of both T2D (OR = 1.91; 95% CI 1.33–2.76, *p* = 0.0005) and MetS (OR = 2.37; 95% CI 1.39–4.05, *p* = 0.0015). Insulin resistance plays a key role in the pathogenesis of T2D.

Unfortunately, there are no other reports yet of an association between the *HSD11B1* rs1208663 polymorphism and insulin resistance (or syndromes with insulin resistance). However, this is slightly less significant than the presence of an association between the presence of the GG rs846910 genotype. The number of individuals in the study population with the GG rs846910 genotype is approximately 90%, whereas the number of individuals with the GG rs12086634 genotype is less than 5%. We would like to emphasize that all individuals with the GG rs12086634 genotype also had the GG rs846910 genotype.

The other two polymorphisms of the *HSD11B1* gene studied (rs4844880, rs3753519), showed no significant association with the occurrence of insulin resistance.

The obtained results of the study are very interesting and bring much new. They performed well in subjects with the GG genotype of rs846910 compared to the AG and AA genotypes of rs846910, and this was true in all the groups studied except for two groups—women and men without insulin resistance. A similar finding holds for subjects with the GG rs1208663 genotype compared to those with the AG and AA rs1208663 genotypes and this was true in all groups except those with insulin resistance. The results of our study suggest that the determination of rs846910 or rs12086634 polymorphism of the *HSD11B1* gene may in the future find practical application in subjects with suspected insulin resistance.

Selective *HSD11B1* blockers have been shown to reduce glucocorticoid production, accelerate weight loss, improve glucose tolerance and insulin sensitivity [28,29,30]. Given the high prevalence of insulin resistance in subjects, studies will be needed to evaluate the effects of these blockers in subjects with different *HSD11B1* gene variants.

## 6. Conclusions

1. There were no statistically significant differences in the distribution of genotypes and alleles of the examined polymorphisms of the 11β-hydroxysteroid dehydrogenase type 1 gene between subjects with and without insulin resistance (determined using the HOMA-IR;
2. There were no statistically significant differences in the distribution of genotypes and alleles of the studied polymorphisms of the 11β-hydroxysteroid dehydrogenase type 1 gene between men and women with and without insulin resistance (determined using the HOMA-IR;
3. rs846910 and rs1208663 polymorphisms of the 11β-hydroxysteroid dehydrogenase type 1 gene in the examined subjects have a significant effect on the magnitude of the HOMA-IR insulin resistance index.

## Figures and Tables

**Table 1:** Basic information about the subjects.

	All Groups	All Women	All Men	All Groups Insulin Resistance	All Group Non-Insulin Resistance Group	Women Insulin Resistance Group	Women Non-Insulin Resistance Group	Men Insulin Resistance Group	Men Non-Insulin Resistance Group
N	507 100%	284 56%	223 44%	374 74%	133 26%	219 77%	65 23%	160 72%	63 28%
Age [year]	52.6 ± 2.3	53 ± 2.9	53 ± 2.7	54.5 ± 3.1	52.2 ± 2.1	54.1 ± 2.9	53.1 ±2.4	54.4 ± 2.8	52.7 ± 2.3
Sex F/M	284/223	284/0	223/0	167/213	56/71	167/0	56/0	0/213	0/71
BMI [kg/m^2^]	26.7 ± 1.3	26.3 ± 1.2	26.6 ± 1.3	27.7 ± 2.3	25.9 ± 0.7	26.7 ± 1.3	25.8 ± 0.9	26.6 ± 1.3	26.0 ± 1.1
Waist [cm]	94.0 ± 4.7	93.1 ± 3.4	95.7 ± 4.1	98.2 ± 4.7	92.1 ± 4.2	95.1 ± 4.4	90.4 ± 4.2	99.7 ± 4.0	94.6 ± 4.8
Hypertension present yes/no	262/245	146/138	133/90	198/176	69/64	111/108	35/30	95/65	38/25
DM present yes/no	52/455	40/244	12/211	29/345	23/110	22/197	18/47	7/153	5/58
eGFR [mL/min/1.73 m^2^]	92 ± 31.2	91 ± 31.2	92 ± 31.2	90 ± 31.1	83 ± 30.4	92 ± 31.2	84 ± 30.5	91 ± 31.4	82 ± 30.5
TC [mmol/l]	6.2 ± 2.0	6.2 ± 1.9	6.3 ± 2.0	6.4 ± 2.0	6.2 ± 1.9	6.3 ± 2.0	6.2 ± 1.8	6.4 ± 2.0	6.2 ± 1.9
HDLC [mmol/l]	1.3 ± 0.4	1.4 ± 0.5	1.4 ± 0.5	1.4 ± 0.5	1.3 ± 0.4	1.3 ± 0.3	1.3 ± 0.4	1.3 ± 0.5	1.2 ± 0.4
LDLC [mmol/l]	4.3 ± 0.7	4.1 ± 0.6	4.3 ± 0.09	4.2 ± 0.7	4.1 ± 0.7	4.3 ± 0.8	4.1 ± 0.8	4.4 ± 0.5	4.2 ± 0.6
TG [mmol/l]	1.4 ± 0.7	1.4 ± 0.7	1.5 ± 0.6	1.5 ± 0.2	1.3 ± 0.4	1.4 ± 0.5	1.3 ±0.4	1.6 ± 0.7	1.4 ± 0.3
HOMA-IR	2.7 ± 1.8	2.5 ± 2.7	2.7 ± 2.6	3.2 ± 0.8	1.1 ± 0.4	3.4 ± 0.5	1.1 ± 0.4	3.1 ± 0.4	1.8 ± 0.4

Abbreviations: BMI-body mass index, DM-diabetes mellitus, eGFR -glomerular filtration rate, TC—total cholesterol, HDLC—cholesterol HDL, LDLC—cholesterol LDL, TG—trugliceride, HOMA-IR—insulin resistance index.

**Table 2:** The distribution of genotypes tested rs12086634, rs846910, rs4844880, rs3753519 of 11β-hydroxysteroid dehydrogenase type 1 (*HSD11B1*) gene in the entire study group of subjects, in women with and without insulin resistance, and in men with and without insulin resistance.

	rs846910			rs3753519			rs4844880			rs1208663		
	AA	AG	GG	CC	CT	TT	AA	AT	TT	TT	GT	GG
All groups N/%	1/0.20	46/9.07	460/90.73	380/74.95	110/21.69	17/3.36	38/7.5	138/27.21	331/65.29	347/68.45	137/27.02	23/4.53
All groups IR N/%	1/0.25	37/9.46	354/90.28	277/75.27	82/21.19	13/3.54	30/8.15	101/26.57	249/65.52	259/66.66	94/28.87	17/4.47
All groups non-IR N/%	0/0	9/7.82	106/92.18	103/76.97	28/20.14	4/2.89	8/6.30	37/29.13	82/64.57	88/61.11	43/34.12	6/4.77
All women	0/0	24/8.46	260/91.54	217/72.98	58/23.58	9/3.44	20/7.04	78/27.46	186/65.5	190/65.56	79/28.93	15/5.49
All men N/%	1/0.45	22/9.87	200/89.68	163/77.18	52/19.77	8/3.05	18/8.08	60/26.90	145/65.02	157/64.95	58/31.64	8/3.41
Women—IR N/%	0/0	18/7.86	211/92.13	159/76.07	43/20.57	7/3.36	16/7.30	58/28.48	145/64.22	139/64.61	58/30.46	12/4.93
Women—non-IR N/%	0/0	6/11.32	47/88.68	58/77.33	15/20.00	2/2.67	4/6.15	20/30.70	41/63.15	51/68.92	21/27.16	3/3.92
Men—IR N/%	1/0.62	16/10.0	143/89.38	114/77.38	39/19.59	6/3.03	14/13.20	43/40.57	104/46.23	120/65.18	36/30.32	5/4.50
Men—non- IR N/%	0/0	6/13.84	59/86.16	49/76.56	13/20.31	2/3.13	4/6.45	17/27.42	41/66.13	37/68.41	22/27.39	3/4.20

**Table 3:** The distribution of alleles tested rs12086634, rs846910, rs4844880, rs3753519 of the 11β-hydroxysteroid dehydrogenase type 1 (*HSD11B1*) gene in the entire study group of subjects, in women with and without insulin resistance, and in men with and without insulin resistance.

	rs846910		rs3753519		rs4844880		rs1208663	
	A	G	C	T	A	T	T	G
All groups N/%	47/4.73	966/95.27	870/85.80	144/14.20	214/21.10	800/78.90	831/81.95	183/18.05
All groups—insulin resistance N/%	38/4.86	745/95.14	636/85.86	108/14.14	161/21.18	599/78.82	612/82.70	128/17.30
All goups–non-insulin resistance N/%	9/3.91	221/96.01	234/86.67	36/13.33	53/20.86	201/79.14	219/79.92	55/20.08
All women N%	24/4.18	544/95.82	492/86.62	76/13.38	118/20.71	450/79.29	459/80.80	109/19.20
All men N/%	23/5.02	422/94.98	378/84.76	68/15.24	96/21.52	350/78.48	372/83.40	74/16.60
Women—IR N/%	18/3.90	440/96.10	361/71.92	141/28.08	90/21.62	348/78.37	336/80.38	82/19.62
Women—non-IR N/%	6/5.45	100/94.55	131/87.33	19/12.66	28/21.53	102/78.46	123/82.00	27/18.00
Men—IR N/%	17/6.30	302/93.70	267/87.18	51/12.81	71/22.19	251/77.81	276/85.71	46/14.28
Men—non-IR N/%	6/4.68	124/95.32	111/86.71	17/13.28	25/20.16	99/79.82	96/77.41	28/22.58

**Table 4:** The mean HOMA-IR values for the different polymorphisms of the type 1 *HSD11B1* gene in the entire study group of subjects, in women with and without insulin resistance, and in men with and without insulin resistance.

	rs846910				rs3753519				rs4844880				rs1208663			
	AA	AG	GG	ANOVA	CC	CT	TT	ANOVA	AA	AT	TT	ANOVA	TT	GT	GG	ANOVA
All groups	2.25 NA	2.66 ± 1.5	2.66 ± 1.5	NS	2.77 ± 0.11	2.65 ± 2.02	2.92 ± 1.62	NS	2.75 ± 1.48	2.77 ± 2.45	2.74 ± 1.98	NS	2.67 ± 1.63	2.82 ± 2.7	3.5 ± 3.16	*p* = 0.042
All groups—insulin resistance	2.2 NA	2.7 ± 1.3	2.8 ± 2.2	*p* = 0.049	3.3 ± 2.2	3.2 ± 2.0	3.4 ± 1.5	NS	3.2 ± 1.3	3.3 ± 2.6	3.3 ± 2.0	NS	3.2 ± 1.6	3.5 ± 2.9	4.5 ± 3.3	*p* < 0.049
All groups—non-insulin resistance	0 NA	1.06 ± 0.4	1.12 ± 0.3	*p* = 0.038	1.13 ± 0.3	1.02 ± 0.3	1.2 ± 0,1	NS	1.06 ± 0.4	1.09 ± 0.6	1.13 ± 0.3	*p* = 0.076	1.13 ± 0.3	1.1 ± 0,3	0.97 ± 0.3	*p* = 0.047
All—women	0 NA	2.88 ± 0.88	2.9 ± 0.37	*p* = 0.048	2.99 ± 0.51	2.46 ± 0.43	3.24 ± 0.9	*p* = 0.048	2.98 ± 0.56	2.77 ± 0.62	2.93 ± 0.28	NS	1.52 ± 0.98	2.97 ± 0.95	3.91 ± 3.7	*p* = 0.034
All—men	2.25 NA	2.42 ± 0.91	2.59 ± 1.81	*p* = 0.049	2.46 ± 1.43	2.85 ± 2.51	2.55 ± 1.25	*p* = 0.049	2.45 ± 1.36	2.76 ± 2.27	2.47 ± 1.42	NS	1.15 ± 0.76	2.8 ± 2.32	2.72 ± 1.69	*p* = 0.044
Women—IR	0 NA	3.56 ± 1.7	3.44 ± 1.4	*p* = 0.049	3.53 ± 2.6	3.06 ± 1.2	4.14 ± 1.7	*p* = 0.049	3.51 ± 1.4	3.41 ± 2.7	3.47 ± 2.3	*p* = 0.047	1.4 ± 1.4	3.59 ± 3.1	4.69 ± 3.7	*p* = 0.023
Women—non-IR	0 NA	0.84 ± 0.3	1.03 ± 0.3	NS	1.03 ± 0.3	0.9 ± 0.3	1.3 ± 0.9	NS	1.11 ± 0.2	0.93 ± 0.3	1.04 ± 0.3	NS	0.12 ± 0.4	0.97 ± 0.4	0.81 ± 0.2	*p* = 0.047
Men—IR	2.25 NA	2.65 ± 0.2	3.2 ± 0.9	*p* = 0.049	3.04 ± 1.4	3.43 ± 1.7	2.96 ± 1.1	*p* = 0.038	2.87 ± 1.2	3.28 ± 1.4	3.08 ± 1.4	*p* = 0.017	3.03 ± 1	3.33 ± 1.6	3.67 ± 1.4	*p* = 0.027
Men—non- IR	0 NA	1.39 ± 0.22	1.2 ± 0.31	NS	1.22 ± 0.3	1.17 ± 0.4	1.27 ± 0.2	*p* = 0.049	1 ± 0.5	1.25 ± 0.3	1.22 ± 0.3	NS	2.22 ± 0.3	1.23 ± 0.3	1.12 ± 0.3	*p* = 0.029

## Data Availability

Not applicable.
